# Disparities in Place of Death Among Malnourished Individuals in the United States

**DOI:** 10.7759/cureus.55503

**Published:** 2024-03-04

**Authors:** Maithili Chamanoor, Riyam Kaur Juneja, Syed Sami, Shamsul Arefin, Daniah Al-Sabbagh, Akhila N Thota, Amna Bint I Munir, Manaswini Chowdary Kaka

**Affiliations:** 1 Internal Medicine, Zhengzhou Medical University, Zhengzhou, CHN; 2 Internal Medicine, Dr. D. Y. Patil Medical College, Hospital, and Research Centre, Pune, IND; 3 Internal Medicine, Karachi Medical and Dental College, Karachi, PAK; 4 Internal Medicine, Nottingham University Hospitals National Health Service (NHS) Trust, Nottingham, GBR; 5 Internal Medicine, University of Baghdad Al-Kindy College of Medicine, Baghdad, IRQ; 6 Medicine, Alluri Sitaram Raju Academy of Medical Sciences, Eluru, IND; 7 Medicine, Fatima Jinnah Medical University, Lahore, PAK; 8 Internal Medicine, American International Medical University, Gros Islet, LCA

**Keywords:** cdc wonder database, end-of-life care, malnutrition, palliative care, home care, hospice care, healthcare disparities, death, mortality trends

## Abstract

Background: Deficiencies or imbalances in a person's intake of nutrients are referred to as malnutrition. Malnutrition remains a significant public health concern in the United States, with potential consequences ranging from chronic disease to mortality. This study aims to assess the disparities in place of death due to malnutrition in the United States from 1999 to 2020, based on variables like age, gender, race, and location, utilizing the Centers for Disease Control and Prevention Information and Communication Wide-Ranging Online Data for Epidemiologic Research (CDC WONDER) database.

Methodology: Data regarding mortality due to malnutrition was extracted for the years 1999-2020 from the CDC WONDER database. Univariate regression analysis was performed to investigate disparities in the place of death based on variables.

Results: Between 1999 and 2020, a total of 1,03,962 malnutrition-related deaths were recorded, with 31,023 in home and hospice care, 68,173 in medical and nursing facilities, and 4,766 in other places. The odds of death due to malnutrition at home or hospice were highest for the 85+ age group, female gender, census region 4 (West), and Asian or Pacific Islander race.

Conclusions: This study reveals a rising trend in mortality due to malnutrition in the United States, especially among certain demographic groups and in medical facilities and nursing homes. It emphasizes the need to understand the factors contributing to this increase in mortality rates. Future research should focus on these contributors to combat the rising burden of malnutrition-related mortality in the United States.

## Introduction

Malnutrition is a condition that is defined by a lack of nutrition, an imbalanced consumption of essential nutrients, or inadequate nutrient utilization. It is caused by a nutrient deficiency that can arise from starvation, a natural disaster, advanced disease, inflammation brought on by disease, or problems with food absorption [1]. Malnutrition is common, but there are no solid diagnostic criteria, which makes it hard to identify malnutrition in hospitals and communities. Nowadays, it is considered a global concern due to its consequences, which include the worsening of underlying diseases and mortality [2].

The number of malnutrition fatalities in the United States hit 6,762 in 2020, or 0.27% of all deaths, according to the most recent WHO data. The death rate, adjusted for age, is 0.90 per 100,000 people. [3] According to the United States Centre for Disease Control and Prevention (CDC), the number of malnutrition-related deaths increased by more than twofold, from around 9,300 in 2018 to about 20,500 in 2022 [3].

Although, in the United States, malnutrition prevalence is relatively low [4], patients' chances of getting malnourished can be increased by several factors, including underlying comorbidities, hospital/nursing facility admission, especially in the elderly, and the various levels of care being provided in these different settings. Nursing homes and hospitals are able to offer patients greater medical care, especially when it comes to end-of-life care, including alleviating their symptoms or providing palliative care. However, home and hospice care are not that efficient.

This study aims to assess the disparities in the number of deaths due to malnutrition in the United States from 1999 to 2020. The objectives include (1) analyzing trends in mortality due to malnutrition across different settings, such as home/hospice care, medical facilities/nursing homes, and other locations; (2) investigating demographic disparities in place of death based on variables such as age, gender, race, and census region; (3) exploring potential factors contributing to the observed disparities in mortality; and (4) discussing the implications of the findings for understanding and addressing malnutrition-related mortality in the United States.

## Materials and methods

Study design

A retrospective study was conducted in September 2023 using a freely accessible, virtually available Centers for Disease Control and Prevention Information and Communication Wide-Ranging Online Data for Epidemiologic Research (CDC WONDER) database, which offers a variety of public health statistics for the United States. The database is searchable and provides age, sex, race, US census region, and cause of death for those patients, as well as information on the site of death and patient-specific demographics whose demise was reported to the CDC by the healthcare provider [5].

Inclusion and exclusion criteria

The CDC WONDER database was utilized to examine malnutrition-related deaths from 1999 to 2020. The selected parameters were the underlying causes of death in the bridged race category within this timeframe. The cause of death was determined using the International Classification of Diseases (ICD) 10th version (ICD-10) classification, with malnutrition (ICD-10 code: E40-E46) chosen as the focus. The study encompassed several variables: age group categorized into ten-year intervals, gender encompassing all sexes, race comprising all races, and census regions covering all US census areas (Northeast, Midwest, South, and West).

The place of death was categorized into three groups: (1) facilities providing skilled medical care were designated as "medical/nursing facility," including medical facility-inpatient, medical facility-outpatient, medical facility-dead on arrival, medical facility-status unknown, and nursing home; (2) facilities offering less skilled medical care were classified as "home/hospice," incorporating the decedent's home and hospice facility; and (3) others, encompassing cases where the place of death was unknown.

Suppressed data (having a value of 9 or less) was excluded from the study, as per the guidelines on the CDC WONDER website to prevent attempts at identification.

Statistical analysis

The statistical analysis was done using univariate logistic regression, which used all the variables from the data to further evaluate disparities in places of death depending on the demographic factors of the patient. Estimated odd ratios (OR) and associated 95% confidence intervals (CI) were achieved. All statistical analyses were evaluated using STATA version 15 (StataCorp LLC, Texas, USA) and R version 3.6 software (The R Foundation, Indiana, USA).

## Results

Aggregate data of 103,962 from 1999-2020 was obtained for malnutrition from the CDC WONDER database. Table 1 shows the total number of deaths due to malnutrition in homes, medical facilities, and other places. The maximum number of deaths in homes and hospices based on age trends is in the age group of 85 years and older and the minimum in five to 14 years. In the case of a medical facility or nursing home, the minimum number of deaths reported was in the age group of one to four years and a maximum of 85 years and above. In other places, no deaths were reported at ages less than 24 years, while the maximum number of deaths occurred in the age group of 85 years and older. Taking gender into account, the number of deaths among females was higher than that of males in all the places. The highest number of deaths occurred among females and in medical facilities, and the minimum number of deaths were reported among males in other places. Based upon the census region, the maximum deaths occurred in census region 2 (Midwest) in the medical facility, and the minimum deaths occurred in the Northeast region, in other places.

**Table 1:** Total number of deaths due to malnutrition in homes, medical facilities, and other places The data is presented as absolute number N

Variables		Home or hospice (n=31023)	Medical facility or nursing (n=68173)	Others (n=4766)
10-year age groups	<1 year	20	104	0
	1-4 years	14	27	0
	5-14 years	0	29	0
	15-24 years	47	94	0
	25-34 years	115	268	15
	35-44 years	236	731	28
	45-54 years	610	1941	77
	55-64 years	1496	4622	196
	65-74 years	3197	9038	364
	75-84 years	7318	18717	1044
	85+ years	17957	32576	3026
Gender	Female	19848	42478	3198
	Male	11175	25695	1568
Census region	Census region 1: Northeast	3131	9134	324
	Census region 2: Midwest	6993	17778	1007
	Census region 3: South	13992	29737	2052
	Census region 4: West	6907	11519	1375
Race	American Indian or Alaska Native	183	543	33
	Asian or Pacific Islander	766	1212	107
	Black or African American	3349	9213	407
	White	26725	57195	4217

Table 2 shows predictors of home or hospice death due to malnutrition from 1999-2020. The odds of death due to malnutrition at home or hospice were highest in the 85+ age group, female gender, and census region 4 (West and Asian Pacific Islanders). Based on age categorization, 85+ years was taken as the reference range, and compared to that, patients of the same age group had the highest odds of death at home or hospice compared to patients of other age groups. No deaths were reported among the patients in the age group of 5-14 years. Considering the groups based on gender, males had a lower likelihood of dying in home or hospice care compared to females by 0.943. Taking census region 1 (Northeast) as the reference number, patients from census region 4 (West) had the highest odds of dying compared to region 1 by 1.618 times. Among the races, patients of the Asian or Pacific Islander race had 1.335 times the chances of dying at a hospice, which was the highest, compared to the white race, which was taken as the reference age.

**Table 2:** Predictors of home or hospice death due to malnutrition from 1999 to 2020 Univariate logistic regression is used. P-value <0.05 is significant

Variables		Univariate logistic regression		
		Odds ratio	95% CI	p-value
Age	<1 year	0.381	(0.236, 0.615)	<0.001
	1-4 years	1.028	(0.539, 1.961)	0.933
	5-14 years	0	(0, 2.55681195748415e+26)	0.766
	15-24 years	0.991	(0.698, 1.408)	0.961
	25-34 years	0.806	(0.648, 1.001)	0.051
	35-44 years	0.616	(0.532, 0.714)	<0.001
	45-54 years	0.599	(0.546, 0.657)	<0.001
	55-64 years	0.616	(0.579, 0.654)	<0.001
	65-74 years	0.674	(0.645, 0.704)	<0.001
	75-84 years	0.734	(0.711, 0.758)	<0.001
	85+ years	1.0 (reference)		
Gender	Male	0.943	(0.918, 0.97)	<0.001
	Female	1.0 (reference)		
Census Region	Census region 1: Northeast	1.0 (reference)		
	Census region 2: Midwest	1.125	(1.071, 1.181)	<0.001
	Census region 3: South	1.33	(1.271, 1.391)	<0.001
	Census region 4: West	1.618	(1.539, 1.701)	<0.001
Race	American Indian or Alaska Native	0.73	(0.618, 0.863)	<0.001
	Asian or Pacific Islander	1.335	(1.219, 1.46)	<0.001
	Black or African American	0.8	(0.767, 0.834)	<0.001
	White	1.0 (reference)		

**Figure 1:**
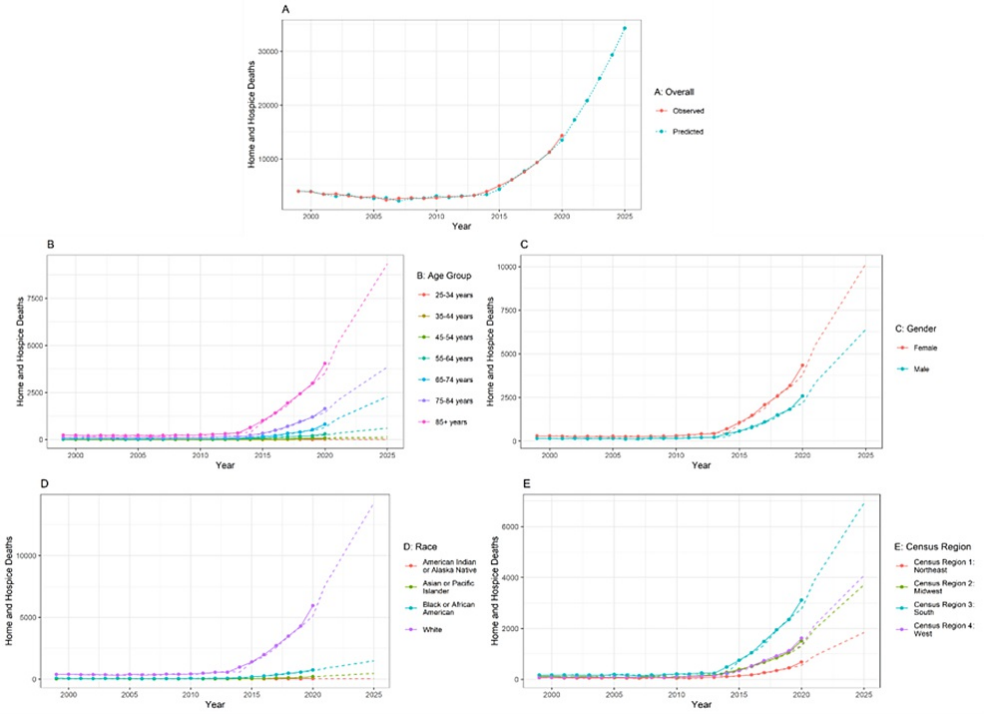
Cumulative home or hospice death trends in case of malnutrition from 1999-2020 The forecasting for A is done from the year 1999 to 2025. The training data is available from the year 1999 to 2020. So, the prediction is done for another five years. In the line chart, the lines represent the observed data. The dotted line represents the forecasted data. The statistical method used for forecasting is the autoregressive integrated moving average (ARIMA) model

Figure 1 shows cumulative home or hospice death trends in cases of malnutrition from 1999-2020. Figure 1A shows the overall and predicted home and hospice deaths in patients with malnutrition. There is a gradual increase in the number of both overall deaths and predicted deaths from 2000 to 2025. In Figure 1B, there is a gradual increase in the number of deaths in all age groups. The maximum number of deaths in home and hospice care was reported in the age groups of 85 years and older. In the case of gender, females recorded a higher number of deaths compared to males. In Figure 1E, all the regions showed a rise in the number of deaths, and the maximum number of deaths was reported in census region 3 (South). Considering the race, there was a significant increase in the number of deaths in patients of the White race compared to the other races.

## Discussion

In this study, the authors identified a rising trend in mortality due to malnutrition in the United States, especially among certain demographic groups and in medical facilities and nursing homes. The findings paint a picture of mortality trends rising in medical/nursing facilities compared to home/hospice care centers. This review offers a systemic look at the study of mortality trends for malnutrition in the United States. The current study demonstrates that malnutrition always remains an important issue for any sort of individual, irrespective of age, gender, race, and region. The main cause of malnutrition is reduced dietary intake. Sadly, malnutrition is a more common cause of death even in developed countries like the United States [6].

When it comes to the mortalities caused by malnutrition, statistical data has been created, and the studies included in the meta-analysis show that there were 31,023 deaths in home or hospice care centers and 68,173 deaths in medical facilities or nursing facilities, and deaths in other places were almost 4,766. This indicates facility differences in susceptibility to malnutrition. The reasons provided for these differences might vary.

When stratified by age groups, the results showed that in homes or hospice centers, reported deaths were highest in individuals more than 85 years of age (17,957), and the death count in individuals living in medical facilities or nursing facilities was also seen with more than 85 years of age (32,576), and the second most common age group was between 75 and 84 years for both home/hospice care and medical/nursing facilities. The primary US community federal nutrition programs state that older adults, regardless of income, must be funded by the US Older Americans Act [7]. The gender of those who suffered from malnutrition compared to those who did not, according to a study conducted by Saunders et al., was no different [6]. The statement in this study shows that there exist differences and that the data occasionally reverses itself, stating that starvation is more common in males than in girls. This study, when stratified on mortality trends of malnutrition based on gender in a particular setting, was collected from the CDC WONDER. It is possible these differences might be underestimated or overestimated. In reviewing the individual studies identified in the main search, results were higher in females living at home/hospice centers compared to males, and females living in medical/nursing facilities have been the gender in reporting the “higher number of deaths.” These preliminary findings might suggest that sex-specific risks vary with age; additional research is necessary. While malnutrition-related deaths have been reported in various northeastern US locations, crude death rates revealed that these deaths are disproportionately more common among residents of medical and nursing facilities. Findings also suggest that access to medical care may not be the best predictor of malnutrition death, but rather the community infrastructure, such as revenue or food availability. Communities with larger proportions of individuals living at home or in hospice care centers were highest among all other settings. In the Midwest region of the United States, reported deaths were highest in individuals living in medical/nursing facilities (17,778). The southern region and western part of the United States also had the highest death reports in medical/nursing facilities (29,737 and 11,519, respectively).

Whites are significantly more likely to be undernourished than Asians, Blacks, or people of other races are [8], but another study by Sadarangani et al. found contradicting findings, with Black people performing among the lowest of all racial groupings in terms of nutrition. In comparison to other races, data on diets with low intakes of fruits, vegetables, and dairy products have remained constant among middle-aged and older Blacks. Communities with higher percentages of Black/African American and Hispanic populations appeared to be less likely to die from malnutrition [9].

However, this study explains that American Indians or Alaska natives living in medical/nursing facilities report a higher number of deaths (543), compared to other settings. Asians or Pacific Islanders (1,212)/Blacks or African Americans (9,213)/Whites (57,195) also reported more deaths in individuals living at medical/nursing facilities. Other studies mention that people living at home, hospice care centers, or other facilities of the above-mentioned race had significantly lower reported deaths compared to those in medical/nursing facilities [10].

According to several other articles [11,12], the study says that malnutrition is an independent predictor. It is highly prevalent in elderly individuals, and it continues to be an important predictor of mortality in elderly people. The above article also mentions the association of COVID-19 with malnutrition in elderly people. Though further validation is required, the unique racial and gender disparities in malnutrition are stated. Adult inpatients with COVID-19 had an increased length of hospital stay and mortality because of malnutrition [12,13].

Nonprofit organizations have the potential to reduce the differences nationally by providing health-sustaining products. Implementing nutritional policies and no longer accepting candy, soda, and sheet cakes will definitely bring about change [14]. Adequate nutrition plays an important role in immune function by inhibiting immune cell activation [15].

Limitations

As seen in various research studies, this epidemiologic study is also subject to certain limitations. Firstly, this study includes statistical data from only the years 1990 to 2020. The study did not include the data from 2021 to 2023, as the current data for these years was not available in the CDC Wonder database. However, through the autoregressive integrated moving average (ARIMA) model, the authors were able to predict the changing trends in the data up to the year 2025. In addition, a second limitation was observed, as the study was not additionally classified into different subcategories. Therefore, the authors maintained a homogenous study of interest at the expense of sub-stratification. However, future research into different subclasses would be pertinent in furthering the understanding of the different trends of death due to malnutrition in the United States. Therefore, recognizing and further evaluating the different mortality trends is pertinent to identifying the underlying factors associated with mortality due to malnutrition.

## Conclusions

Mortality due to malnutrition has been increasingly contributing to the mortality rates in the United States and is only anticipated to rise. Consequently, it is essential to thoroughly understand the contributing factors and trends associated with deaths due to malnutrition. To increase our awareness, it is crucial for future research studies to recognize the underlying contributors to the increased incidence of deaths in medical facilities and nursing homes. It is vital to understand these factors to improve end-of-life care for patients and minimize mortality in medical facilities and nursing homes. By further identifying the mortality trends in malnutrition, we can emphasize its growing burden and strive to reduce its prevalence in the United States.
